# Interaction of spirituality with entrepreneurial attitudes and intentions in South Korea: extending the theory of planned behavior

**DOI:** 10.3389/fpsyg.2026.1756793

**Published:** 2026-04-29

**Authors:** Bernard Lee, Namjae Cho, Oliver H. M. Yau, Giseob Yu

**Affiliations:** 1 CBCC, Saint Francis University, Hong Kong, Hong Kong SAR, China; 2 School of Business, Hanyang University, Seoul, Republic of Korea; 3 College of Business, City University of Hong Kong, Hong Kong, Hong Kong SAR, China; 4 Division of Interdisciplinary Studies for International Students, Sun Moon University, Asan, Republic of Korea

**Keywords:** entrepreneurship, entrepreneurial behavior, entrepreneurial intention, extended theory of planned behavior, South Korea

## Abstract

**Context:**

The Theory of Planned Behavior (TPB) is widely applied to explain entrepreneurial intentions and behavior. However, the role of spirituality, understood as a personal, existential search for meaning and connection beyond institutional religious frameworks, remains underexplored, particularly in collectivist Asian contexts where cultural values may interact with spiritual beliefs to shape entrepreneurial pathways.

**Objective:**

This study extends the TPB by examining how spirituality interacts with the three core antecedents of entrepreneurial intention, such as attitude toward entrepreneurship, subjective norms, and perceived behavioral control, and moderates the relationship between entrepreneurial intention and subsequent entrepreneurial behavior in South Korea.

**Method:**

A quantitative survey was conducted with 204 university students and recent graduates in South Korea, representing diverse sectors such as technology, fashion, and food services. The instrument included validated scales for TPB constructs, entrepreneurial intention, preparatory entrepreneurial behavior, and a spirituality measure adapted to the Korean context. Data were analyzed using partial least squares structural equation modeling (PLS-SEM) via SmartPLS.

**Results:**

Findings confirm that the three TPB antecedents significantly predict entrepreneurial intention, and intention, in turn, predicts behavior. Spirituality was found to positively moderate the intention–behavior relationship, suggesting that spiritually inclined individuals are more likely to translate intentions into action. However, its moderating role on the antecedents of intention showed mixed results.

**Implications:**

This study contributes to entrepreneurial behavior research by integrating spirituality into the TPB framework within a non-Western, collectivist setting. It underscores the importance of culturally sensitive yet theoretically grounded extensions of behavioral models and opens avenues for further qualitative and cross-cultural exploration of spirituality in entrepreneurship. The findings are most directly applicable to students and recent graduates in similar cultural contexts; future research should examine generalizability to established entrepreneurs and other institutional settings.

## 1 Introduction

Entrepreneurship plays a crucial role in economic growth and innovation across various industries, contributing to job creation, technological advancement, and social development. The entrepreneurial landscape is characterized by rapid technological change, shifting consumer preferences, increasing diversity, and significant market potential—factors that collectively render it an attractive domain for aspiring entrepreneurs. However, not all opportunities trigger entrepreneurial intention (EI), nor do all intentions translate into entrepreneurial behavior (EB). Understanding the psychological and contextual mechanisms that govern the intention–behavior relationship, therefore, remains a central concern in entrepreneurial research (Ajzen, 1991; Schlaegel and Koenig, 2014).

This study investigates entrepreneurial intention and behavior through the lens of the Theory of Planned Behavior (TPB). Developed by (Ajzen 1985, 1991, 2002), the TPB posits that individual behavior is shaped by three core antecedents: attitude toward the behavior, subjective norms, and perceived behavioral control. The framework has received extensive empirical support in entrepreneurship research, with meta-analyses confirming its predictive validity across diverse contexts (Schlaegel and Koenig, 2014). However, existing research often overlooks the nuanced influence of cultural contexts and deeply held personal belief systems, which may moderate these theoretically postulated relationships. While recent extensions of the TPB have incorporated variables such as emotions, experience, and personality traits (Sonkar et al., 2026), the role of spirituality, dimension of personal meaning, transcendence, and value-driven orientation remains underexplored in entrepreneurial scholarship.

Spirituality, defined as a personal, existential search for meaning, purpose, and connection that extends beyond institutional religious frameworks (Kriger and Seng, 2005), has been shown to provide a transcendent source of motivation that aligns entrepreneurial action with deeper personal and social values (Rashid and Ratten, 2022; Dissanayake et al., 2025). Unlike religiosity, which refers to adherence to organized religious doctrines and practices, spirituality encompasses a broader set of existential concerns, including meaning-making, inner peace, and a sense of interconnectedness with others and the natural world (Zinnbauer et al., 1997; Fisher, 2021). This distinction is critical, as individuals may identify as non-religious while still endorsing spiritual beliefs that shape their values, decision-making, and motivation.

From the perspective of Cognitive-relational Theory (Lazarus and Folkman, 1984), spiritual beliefs serve as coping resources that reframe entrepreneurial challenges, such as failure, uncertainty, and resource constraints, as meaningful opportunities for growth or tests of character. Additionally, Social Identity Theory (Tajfel and Turner, 2001) suggests that spiritual communities function as salient reference groups that shape subjective norms and provide social support for entrepreneurial action. Despite this growing recognition of spirituality's potential relevance, little empirical research has systematically integrated spirituality into the TPB framework to examine its specific influence on the entrepreneurial intention and behavior pathway. The present study addresses this gap by extending the TPB to incorporate spirituality as both a moderating factor and an antecedent within a culturally distinct context.

**Figure 1:**
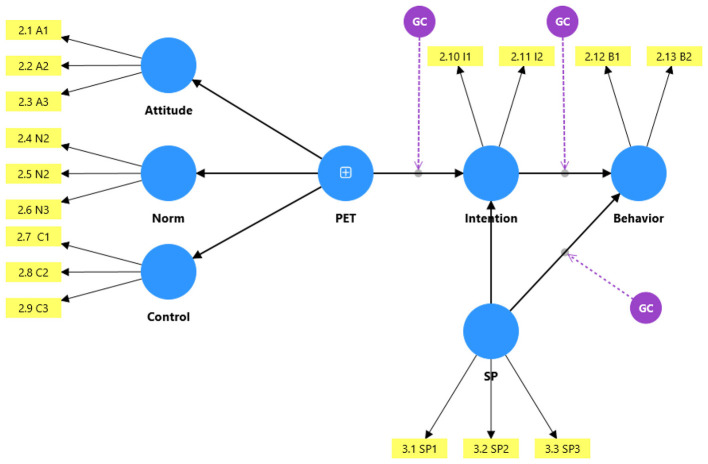
Planned Entrepreneurial Trait (PET) Model.

The theoretical model guiding this investigation, referred to as the Extended TPB with Spirituality, is presented in Figure 1. The model conceptualizes Planned Entrepreneurial Traits (PET) as a second-order construct comprising the three traditional TPB antecedents (attitude, subjective norms, and perceived behavioral control). We posit that PET directly influences entrepreneurial intention, which in turn predicts entrepreneurial behavior. Critically, spirituality is hypothesized to moderate two key pathways: (a) the relationship between PET and entrepreneurial intention, and (b) the relationship between entrepreneurial intention and entrepreneurial behavior. By operationalizing PET as a higher-order construct, the model captures the synergistic effect of the three TPB attributes while allowing for the examination of moderating effects. This study addresses the following research question:

*How does spirituality influence the formation of entrepreneurial intention and its translation into entrepreneurial behavior within the Extended Theory of Planned Behavior framework?*

In answering this question, we make a unique contribution relative to prior work. Unlike (Sonkar et al. 2026), who extended the TPB by incorporating personality traits without cultural contextualization, the present study explicitly examines spirituality as a theoretically grounded moderator that operates through mechanisms of meaning-making, resilience, and social identification. This approach responds to calls for greater attention to context-specific psychological mechanisms in entrepreneurial behavior research (Welter, 2011) and advances theoretical understanding of how personal belief systems interact with planned behavioral processes.

We examine the proposed model in South Korea, a context that offers distinct advantages for studying spirituality in entrepreneurship. The Korean entrepreneurial landscape is characterized not only by rapid technological advancement and strong institutional support but also by a distinct cultural-spiritual fabric that shapes individual cognition and behavior. Core cultural constructs such as *jeong* (deep relational attachment) and *han* (a collective sense of resilient perseverance), rooted in Confucian and Buddhist traditions, create a unique environment where personal belief systems interact with social expectations (Cho, 2009). These culturally specific spiritual expressions can be understood as local manifestations of the broader spiritual mechanisms—relational connection, meaning-making, and resilience—theorized above. By testing our model in this context, we aim to clarify how spirituality influences entrepreneurial pathways, while also considering the potential boundary conditions for applying these findings to other collectivist or spiritually oriented cultures.

The remainder of this paper is structured as follows. Section 2 presents the literature review and hypothesis development, beginning with an examination of spirituality and entrepreneurship, followed by the theoretical framework, and culminating in the formulation of seven research hypotheses. Section 3 describes the research methodology, including research design, participants, procedure, measures, and data analysis techniques. Section 4 reports the empirical findings, structured according to measurement model assessment and structural model evaluation. Section 5 discusses the results, organized into a summary of findings, theoretical implications, practical implications, limitations, and directions for future research.

## 2 Literature review

### 2.1 Spirituality and entrepreneurship

Spirituality in the entrepreneurial context represents a personal, holistic search for meaning, purpose, and connection that extends beyond formal religious adherence (Kriger and Seng, 2005; Moya and Toledano, 2025). This intrinsic belief system shapes core values, ethical reasoning, and resilience—critical psychological resources for navigating venture creation (Shepherd et al., 2024). Unlike religiosity, which refers to adherence to organized religious doctrines and practices, spirituality encompasses a broader set of existential concerns, including meaning-making, inner peace, and a sense of interconnectedness with others and the natural world (Zinnbauer et al., 1997; Fisher, 2021).

Contemporary research underscores that spirituality can act as a foundational resource, providing a transcendent source of motivation that aligns entrepreneurial action with deeper personal and social values, thereby supporting business models that integrate profit with purpose (Rashid and Ratten, 2022; Rosales and Silveyra, 2024). This alignment fosters a form of value-driven entrepreneurship where ventures are often viewed as expressions of personal calling or social contribution (Singh and Awasthy, 2025).

From the perspective of Cognitive-relational Theory (Lazarus and Folkman, 1984), spiritual beliefs serve as coping resources that reframe entrepreneurial challenges, such as failure, uncertainty, and resource constraints, as meaningful opportunities for growth or tests of character. Additionally, Social Identity Theory (Tajfel and Turner, 2001) suggests that spiritual communities, including religious congregations, temple networks, and mindfulness groups function as salient reference groups that shape subjective norms and provide social support for entrepreneurial action (Balog et al., 2014). Membership in such communities offers both normative encouragement and tangible resources, thereby facilitating venture creation and persistence.

To contextualize this study within South Korea, it is essential to consider how these general spiritual mechanisms manifest in culturally specific forms. The Korean entrepreneurial landscape is characterized by a distinct cultural-spiritual fabric that shapes individual cognition and behavior. Core cultural constructs such as *jeong* (deep relational attachment) and *han* (a collective sense of resilient perseverance), rooted in Confucian and Buddhist traditions, create a unique environment where personal belief systems interact with social expectations (Cho, 2009). *Jeong* represents a culturally specific expression of the relational connectedness emphasized in Social Identity Theory, while *han* reflects a culturally situated form of resilience consistent with the cognitive reappraisal mechanisms described in Cognitive-relational Theory.

Despite these insights and the growing body of international work (Fisher, 2021; Rosales and Silveyra, 2024), notable research gaps persist. Much of the existing literature relies on measurement tools developed in Western contexts (Paloutzian and Ellison, 1982). Furthermore, while qualitative and case-study research is emerging, there remains a scarcity of large-scale quantitative studies that systematically model spirituality as an integrated variable within established theoretical frameworks like the Theory of Planned Behavior (TPB) (Kautonen et al., 2015). This study aims to address this gap by developing a context-sensitive model that builds on contemporary spirituality-entrepreneurship research to empirically test how spirituality functions both as an antecedent and a moderating factor within the entrepreneurial intention–behavior pathway in South Korea.

Before concluding this section, it is important to acknowledge that spirituality is not universally beneficial in the entrepreneurial context. Emerging research has begun to document potential “dark side” effects, including the risk of spiritual bypass, where individuals use spiritual beliefs to avoid confronting practical business challenges, and excessive rigidity in adhering to spiritually derived principles that may impede necessary adaptation (Shepherd et al., 2024). In the Korean context, the intense commitment demanded by certain spiritual or religious communities could potentially exacerbate burnout or create conflicts between entrepreneurial demands and spiritual obligations. While the present study focuses on the positive moderating role of spirituality, we acknowledge these boundary conditions as important considerations for future research and for interpreting the scope of our findings.

### 2.2 Theoretical framework

The Theory of Planned Behavior (TPB), initially developed by (Ajzen 1985, 1991, 2002), provides a psychological framework for analyzing how behavioral intentions are influenced by three foundational constructs: attitude toward the behavior, subjective norms, and perceived behavioral control. Attitude reflects an individual's evaluation of the desirability of engaging in entrepreneurship, weighing perceived benefits against potential risks (Kautonen et al., 2015). Subjective norms represent the perceived social pressures from family, peers, and society, which are particularly influential in collectivist cultures like South Korea (Liñán and Chen, 2009). Perceived behavioral control, closely related to self-efficacy, pertains to an individual's confidence in their ability to execute entrepreneurial tasks, shaped by access to resources and relevant skills (Ajzen, 2002). Meta-analytic evidence confirms that these three antecedents collectively explain a substantial proportion of variance in entrepreneurial intention, with average effects ranging from moderate to strong across diverse cultural contexts (Schlaegel and Koenig, 2014).

Research has expanded the TPB to include additional variables to improve its explanatory and predictive power across diverse settings. These extensions are often context-driven, incorporating factors relevant to specific populations or environments. For instance, studies have applied the extended TPB to examine the role of entrepreneurship education and academic major in shaping intentions (Solesvik, 2013), to investigate the influence of personal values and contextual factors in developing countries (Karimi et al., 2017), and to assess the impact of educational support alongside personality traits (Maheshwari, 2021). Other applications have integrated constructs such as prior entrepreneurial experience, risk perception, and access to finance to better understand entrepreneurial intention in various cultural and institutional settings (Estrin et al., 2008).

Building on this tradition of extension, this study first introduces the construct of Planned Entrepreneurial Traits (PET). PET is conceptualized as a reflective, higher-order construct comprised of the three primary TPB dimensions, i.e., entrepreneurial attitude, subjective norms, and perceived behavioral control, specifically within the venture creation context (Ajzen, 1991; Lee et al., 2016). The rationale for employing a second-order construct is twofold. First, theoretically, the three TPB antecedents are conceptually distinct yet collectively represent an individual's overall entrepreneurial readiness or predisposition (Schlaegel and Koenig, 2014). Second, methodologically, modeling PET as a higher-order construct allows for the examination of moderation effects while preserving the theoretical integrity of the underlying dimensions (Becker et al., 2012). This approach aligns with recent advancements in TPB research that recognize the synergistic operation of the three antecedents in shaping intention (Liñán and Fayolle, 2015). By integrating spirituality within an extended TPB model that includes PET, this research seeks to offer a more comprehensive understanding of entrepreneurial decision-making informed by Cognitive-relational Theory and Social Identity Theory.

### 2.3 Hypothesis development

In line with the extended TPB, the basic Planned Entrepreneurial Traits (PET) comprise three dimensions: entrepreneurial attitude, entrepreneurial norm (subjective norms), and entrepreneurial control (perceived behavioral control), as conceptualized in Figure 1. Rather than examining the individual effects of each dimension separately, this study examines their collective effect by treating them as indicators of a reflective second-order construct. This approach is consistent with theoretical treatments of the TPB as an integrated motivational system (Ajzen, 1991) and is optimally assessed through confirmatory factor analysis. Accordingly, we propose:

**H1:** Attitude toward entrepreneurship, subjective norms, and perceived behavioral control are three dimensions that form the higher-order construct of Planned Entrepreneurial Trait (PET).

#### 2.3.1 Planned entrepreneurial trait (PET) to intention

Within the TPB framework, the three antecedents collectively shape entrepreneurial intention (Ajzen, 1991). Entrepreneurial attitude—the degree to which an individual evaluates entrepreneurship as desirable—serves as a core predictor; individuals who perceive entrepreneurship as rewarding and fulfilling are more likely to develop strong intentions to initiate business ventures (Liñán and Chen, 2009). Perceived behavioral control captures self-efficacy beliefs; individuals who perceive themselves as capable of executing entrepreneurial tasks demonstrate stronger intentions, particularly when institutional support mechanisms are accessible (Kautonen et al., 2015).

Empirical studies confirm that these three antecedents collectively explain substantial variance in entrepreneurial intention across diverse contexts. Schlaegel and Koenig's (2014) meta-analysis of TPB applications in entrepreneurship reported an average corrected correlation of β = 0.45 for the combined effect of the three antecedents on intention. In South Korea, where cultural values emphasize achievement and self-efficacy, these relationships are particularly pronounced (Lee et al., 2016). Accordingly, we hypothesize:

**H2:** Planned Entrepreneurial Trait (PET) has a significant positive impact on entrepreneurial intention.

#### 2.3.2 Intention as a driver of behavior

Intention is the most immediate antecedent of behavior in the TPB framework (Ajzen, 1991). Given that the sample consists of students and recent graduates who are potential rather than established entrepreneurs, we focus on preparatory entrepreneurial behavior that serve as immediate precursors to venture creation, such as developing a business plan, seeking funding, conducting market research, or networking for the venture (Kautonen et al., 2015). Strong entrepreneurial intentions increase the likelihood of undertaking such concrete preparatory steps.

Meta-analytic evidence indicates that intention explains approximately 28% of the variance in behavior, leaving substantial room for moderating influences (Sheeran, 2002). In the entrepreneurial domain, the intention-behavior link is particularly consequential, as venture creation requires sustained effort over time (Kautonen et al., 2015). In light of this, we formulate:

**H3:** Entrepreneurial intention has a significant positive impact on preparatory entrepreneurial behavior.

#### 2.3.3 The antecedent role of spirituality

The TPB posits that intention is a function of attitude, subjective norms, and perceived behavioral control. To meaningfully extend this framework, we propose spirituality as an antecedent variable that shapes these core cognitive antecedents, thereby influencing the formation of entrepreneurial intention.

First, spirituality can fundamentally shape an individual's attitude toward entrepreneurship. As a search for meaning and purpose beyond the self (Kriger and Seng, 2005), spirituality can frame entrepreneurship as more than a purely economic endeavor as a vocation, a path to self-actualization, or a means to create meaningful social value (Smith et al., 2023; Rashid and Ratten, 2022). This transcendent motivation can lead to a more positive, resilient, and value-laden evaluation of the entrepreneurial career path, enhancing its perceived desirability.

Second, spirituality influences subjective norms. From a Social Identity Theory perspective (Tajfel and Turner, 2001), spiritual communities constitute salient reference groups whose values and expectations shape individuals' normative beliefs. In collectivist cultures like South Korea, spiritual beliefs are often interwoven with familial and social duties. Furthermore, spiritual communities themselves—religious congregations, temple networks, meditation groups—provide normative support and models for value-driven entrepreneurship (Balog et al., 2014).

Third, spirituality can bolster perceived behavioral control. Consistent with Cognitive-relational Theory (Lazarus and Folkman, 1984), spiritual beliefs and practices, such as meditation or reflective prayer, serve as coping resources that enhance emotional regulation, focus, and resilience (Goyal et al., 2014). This internal fortitude can increase an individual's confidence in their ability to plan, persevere, and overcome the inherent uncertainties and setbacks of the entrepreneurial process (Cho, 2009). The sense of support derived from a spiritual belief system or community can also mitigate feelings of isolation and self-doubt, effectively raising perceived control.

By influencing these three foundational drivers of intention, spirituality serves as an antecedent within the extended TPB framework. Therefore, we posit a direct relationship between spirituality and the strength of entrepreneurial intention:

**H4:** Spirituality has a significant positive impact on entrepreneurial intention.

#### 2.3.4 Behavior driven by spirituality

Following the same rationale, we posit that spirituality may directly influence the likelihood of taking preparatory entrepreneurial actions, independent of its effects through intention. The coping resources provided by spiritual beliefs enable individuals to persist in the face of setbacks (Lazarus and Folkman, 1984). Spiritual entrepreneurs often exhibit greater persistence and ethical consistency, leading to sustained efforts toward business creation (Dodd and Seaman, 1998). Additionally, spiritual networks provide emotional and informational support that can directly facilitate concrete action, irrespective of formally stated intentions (Balog et al., 2014). Accordingly, we propose:

**H5:** Spirituality has a significant positive impact on preparatory entrepreneurial behavior.

#### 2.3.5 Planned entrepreneurial prerequisite enhanced by spirituality

Beyond its direct effects, spirituality may amplify the relationship between the TPB antecedents (captured as PET) and entrepreneurial intention. Through mechanisms of cognitive reappraisal (Lazarus and Folkman, 1984), spirituality can mitigate self-doubt by providing a framework for interpreting potential failure as meaningful rather than catastrophic. Spirituality can mitigate self-doubt by providing a sense of divine support, karmic justice, or cosmic meaning that reduces the psychological weight of potential failure (Balog et al., 2014). Therefore, we propose:

**H6:** Spirituality positively moderates the relationship between Planned Entrepreneurial Trait (PET) and entrepreneurial intention, such that the relationship is stronger for individuals with higher levels of spirituality.

#### 2.3.6 Bridging intention and action

The intention-behavior gap is a well-documented phenomenon in psychological research, with meta-analyses indicating that intention explains only a portion of behavioral variance (Sheeran, 2002). Spirituality may serve as a critical bridging mechanism that helps ensure entrepreneurial intentions translate into preparatory action. Cognitive-relational Theory further suggests that spiritual beliefs enable individuals to interpret setbacks as opportunities for growth rather than as threats, thereby maintaining motivation (Lazarus and Folkman, 1984). Spiritual commitment fosters discipline, long-term vision, and resilience—qualities that support sustained effort toward goal attainment (Shepherd et al., 2024). In Korea, where spiritual entrepreneurs may interpret setbacks as tests of faith or opportunities for character development, they tend to persist in taking concrete steps where others might abandon their efforts (Cho, 2009). Moreover, accountability to spiritual communities can reduce procrastination and increase commitment, facilitating the execution of entrepreneurial plans (Dodd and Seaman, 1998). Consequently, we assert:

**H7:** Spirituality positively moderates the relationship between entrepreneurial intention and preparatory entrepreneurial behavior, such that the relationship is stronger for individuals with higher levels of spirituality.

Table 1 presents the summary of hypotheses examined in the study.

**Table 1:** Summary of research hypotheses.

Hypothesis	Statement	Expected sign	Key supporting references
H1	Attitude, norms, and control form the higher-order PET construct	+	Ajzen, 1991; Lee et al., 2016; Becker et al., 2012
H2	PET → Entrepreneurial Intention	+	Ajzen, 1991; Schlaegel and Koenig, 2014; Liñán and Chen, 2009
H3	Entrepreneurial Intention → Preparatory Behavior	+	Ajzen, 1991; Kautonen et al., 2015; Sheeran, 2002
H4	Spirituality → Entrepreneurial Intention	+	Smith et al., 2023; Rashid and Ratten, 2022; Balog et al., 2014
H5	Spirituality → Preparatory Behavior	+	Dodd and Seaman, 1998
H6	Spirituality moderates PET → Intention	+	Balog et al., 2014
H7	Spirituality moderates Intention → Behavior	+	Shepherd et al., 2024; Sheeran, 2002

## 3 Research method

### 3.1 Research design

This study employed a quantitative survey methodology using a cross-sectional design. Data were collected through a structured questionnaire administered to university students and recent graduates in South Korea. The cross-sectional approach was appropriate for examining the hypothesized relationships among spirituality, TPB constructs, entrepreneurial intention, and preparatory entrepreneurial behavior at a single point in time.

### 3.2 Participants

The target population consisted of university students and recent graduates in South Korea—individuals at a career stage where entrepreneurial pathways are actively considered. A total of 321 questionnaires were distributed, yielding 204 valid responses, representing a response rate of 63.5%. The sample comprised business students and recent graduates from a large university in Seoul, with a mean age of 22.3 years (SD = 2.8). The sample included 112 male respondents (54.9%) and 92 female respondents (45.1%).

To determine the required sample size, we conducted an a priori power analysis using G^*^Power software (Faul et al., 2009). Assuming a medium effect size (*f*^2^ = 0.15), α = 0.05, and power (1–β) = 0.95, the analysis indicated a minimum required sample size of 111. To ensure robustness and account for potential incomplete responses, we established a target sample size of 189. The obtained sample of 204 exceeds both the minimum requirement and the target, confirming adequate statistical power for hypothesis testing.

### 3.3 Procedure

Data collection took place between January and March 2025 at a large university in Seoul, South Korea. Participants were recruited through university mailing lists and invited to complete a paper-based survey. The questionnaire required ~10 min to complete. Prior to participation, all respondents were informed about the purpose of the study, the voluntary nature of participation, and the confidentiality of their responses. Informed consent was obtained from all participants.

To ensure linguistic equivalence, the questionnaire was administered in Korean following a back-translation procedure. The original English items were translated into Korean by a bilingual researcher, then independently back-translated into English by a second bilingual researcher. Discrepancies were discussed and resolved to ensure that the Korean version preserved the meaning of the original items.

### 3.4 Measures

All constructs were measured using established scales with documented psychometric properties. Unless otherwise noted, items were rated on a 6-point Likert scale ranging from 1 (strongly disagree) to 6 (strongly agree). The use of an even-point scale follows recommendations for research in Asian contexts to reduce central tendency bias (Yang, 1982; Yau, 1994).

#### 3.4.1 Theory of planned behavior (TPB) dimensions

The three core TPB antecedents, i.e., attitude toward entrepreneurship, subjective norms, and perceived behavioral control, were measured using items adapted from (Ajzen 1991) and validated in entrepreneurial contexts by (Liñán and Chen 2009). Sample items include: “A career as an entrepreneur is attractive to me” (attitude); “My family values entrepreneurial activity above other career options” (subjective norms); and “I am prepared to start a viable business” (perceived behavioral control).

#### 3.4.2 Entrepreneurial intention

Entrepreneurial intention was measured using the scale developed by (Liñán and Chen 2009), consisting of six items. Sample items include: “I am ready to do anything to be an entrepreneur” and “My professional goal is to become an entrepreneur.” The scale demonstrated excellent reliability in the present study (Cronbach's α = 0.89).

#### 3.4.3 Preparatory entrepreneurial behavior

Because the sample consisted of students and recent graduates rather than established business owners, entrepreneurial behavior was operationalized as preparatory actions toward venture creation. We employed the scale developed by (Kautonen et al. 2015), which assesses concrete behaviors such as business planning, resource seeking, and networking. Sample items include: “I have been gathering information about starting a business” and “I have been discussing my business idea with potential customers or partners.” The scale showed strong reliability (Cronbach's α = 0.92).

#### 3.4.4 Spirituality

Spirituality was measured using a six-item scale developed by (Lee 2024) for the Korean context. Consistent with contemporary definitions that distinguish spirituality from religiosity (Kriger and Seng, 2005; Zinnbauer et al., 1997), the scale assesses existential dimensions of spirituality rather than religious practice. The items capture personal search for meaning (“I believe my life has meaning and purpose”), inner peace (“I maintain my inner peace whatever happens”), acceptance of limited control (“I accept that I am not in full control of the course of my life”), and sense of connection beyond self (“I feel strongly connected when I am in nature”). Sample items include: “I find meaning in my life through spiritual reflection” and “My spiritual beliefs guide my important decisions.” The scale's focus on existential and meaning-based dimensions makes it appropriate for use with non-religious respondents, who may endorse spiritual beliefs while not identifying with organized religion (Fisher, 2021). It should be noted that while this scale has been used in Korean samples, its cultural validation relative to Western spirituality measures requires further confirmation, which is a limitation addressed in Section 5.

#### 3.4.5 Common method variance safeguards

To detect potential common method variance, two marker variables were randomly inserted into the questionnaire as recommended by (Lindell and Whitney 2001). The complete list of measurement items for all constructs is provided in Appendix A.

### 3.5 Data analysis

Data analysis was conducted using SmartPLS version 4.1.1.1 (Ringle et al., 2015). Partial Least Squares Structural Equation Modeling (PLS-SEM) was selected for three primary reasons. First, the study's focus on predicting and explaining key target constructs (entrepreneurial intention and behavior) aligns with PLS-SEM's predictive orientation (Hair et al., 2017). Second, PLS-SEM accommodates the analysis of higher-order constructs, specifically, Planned Entrepreneurial Traits (PET) as a reflective second-order construct. Third, PLS-SEM does not require multivariate normality, making it suitable for the data characteristics encountered in this study (Hair et al., 2019).

The analysis proceeded in two stages: assessment of the measurement model, followed by evaluation of the structural model. Bootstrapping with 5,000 resamples was employed to generate standard errors and confidence intervals for path coefficient estimates (one-tailed testing, α = 0.05).

#### 3.5.1 Validity and reliability tests

##### 3.5.1.1 Measurement model assessment

Reliability was evaluated using Cronbach's alpha and composite reliability (CR), with thresholds of 0.70 or higher indicating adequate reliability (Nunnally, 1978). Convergent validity was assessed through average variance extracted (AVE), with values exceeding 0.50 considered acceptable (Fornell and Larcker, 1981). Discriminant validity was examined using the Heterotrait-Monotrait ratio (HTMT), with values below 0.85 indicating adequate discriminant validity (Henseler et al., 2015).

##### 3.5.1.2 Common method variance (CMV) test

Because data were collected from a single source at one point in time, common method variance poses a potential concern. We employed two approaches to assess CMV. First, Harman's single-factor test was conducted; the unrotated factor solution revealed that the first factor accounted for less than 50% of the variance, suggesting that CMV is not a pervasive issue (Podsakoff et al., 2003). Second, we used the marker variable approach (Lindell and Whitney, 2001), comparing correlations among substantive constructs before and after partialling out the marker variable. The results indicated no substantial inflation of correlations due to common method bias.

##### 3.5.1.3 Power analysis

*Post hoc* statistical power was assessed using the observed *R*^2^ values for the endogenous constructs (entrepreneurial intention and preparatory behavior). Following guidelines by (Cohen 1988), the achieved power exceeded 0.95 for all key relationships, confirming the adequacy of the sample size.

#### 3.5.2 Endogeneity assessment

To strengthen causal inference for the core structural relationships hypothesized in H2, H3, and H5, and to test for potential endogeneity, we employed the Gaussian Copula approach following the procedure outlined by (Park and Gupta 2012). This approach tests whether the identified path coefficients represent causal effects by modeling the correlation between the endogenous predictor variables and the error term. Non-significant Gaussian Copula terms indicate that endogeneity is not a serious concern in the estimated model.

## 4 Findings

### 4.1 Profile of respondents

To obtain relevant data for this study, a structured questionnaire was distributed to undergraduates and recent graduates. Out of 321 surveys disseminated, a total of 204 valid responses were collected, resulting in a response rate of 63.5%. The gender composition of the sample was relatively balanced, with male respondents accounting for 51.5% (*n* = 105) and female respondents comprising 48.5% (*n* = 99). In terms of age, the majority of participants (55.4%, *n* = 113) were between 21 and 25 years old, while ~one-quarter (26%, *n* = 53) were 20 years old or younger (mean age = 22.3 years, SD = 2.8).

Regarding academic background, the sample was predominantly comprised of individuals with undergraduate-level education. Specifically, 73% (*n* = 149) were currently enrolled in or had completed an undergraduate program, whereas 17.2% (*n* = 35) reported enrollment in or completion of graduate-level studies. Household income levels revealed that the most frequently reported income range was between $2,729 and $5,457 per month (32.4%, *n* = 66). A smaller proportion of respondents reported monthly incomes below $2,728 (22.5%, *n* = 46), while 17.6% (*n* = 36) indicated earnings between $5,458 and $8,186. These figures suggest that the sample largely consists of younger individuals from middle-income households.

As for religious affiliation, a significant majority (69.6%, *n* = 142) identified as non-religious. Protestants constituted 17.6% (*n* = 36) of the sample, followed by Buddhists comprising 6.9% (*n* = 14) and Catholics comprising 5.4% (*n* = 11). It is important to note that, consistent with the conceptual distinction between spirituality and religiosity (Zinnbauer et al., 1997; Fisher, 2021), individuals who identify as non-religious may still endorse spiritual beliefs related to meaning-making, inner peace, and connection beyond self. The *post-hoc* robustness check reported in Section 4.3.2 confirms that the effects of spirituality on entrepreneurial intention and behavior are significant even within the non-religious subsample. Table 2 presents an overview of the respondents' demographic characteristics.

**Table 2:** Result of demographic analysis.

Category		Frequency	%
Gender	Male	105	51.47
	Female	99	48.53
Age	20 or Below	53	25.98
	21–25	113	55.39
	26–30	9	4.41
	31–35	6	2.94
	36 or Above	23	11.27
Education	High School	16	7.84
	College or Diploma	4	1.96
	University	149	73.04
	Graduate School	35	17.16
Family income	$2,728 or Below	46	22.55
	$2,729–$5,457	66	33.35
	$5,458–$8,186	36	17.65
	$8,187–$10,914	13	6.37
	$10,915 or Above	25	12.25
	Others	18	8.82
Religion	None	142	69.61
	Protestantism	36	17.65
	Buddhism	14	6.86
	Catholicism	11	5.39
	Islam	1	0.49
Interested industry	Retail	66	32.35
	IT Service	47	23.04
	Education	33	16.18
	Textile and manufacturing	18	8.82
	Cosmetics	13	6.37
	Others	27	13.23
Family influence	Yes	124	60.78
	No	80	39.22

### 4.2 Measurement model assessment

Prior to testing the structural relationships, we assessed the measurement model to establish the reliability and validity of the constructs. This section reports the results of convergent validity, discriminant validity, and reliability tests.

#### 4.2.1 Convergent validity and reliability

Convergent validity was evaluated using average variance extracted (AVE), with values exceeding 0.50 considered acceptable (Fornell and Larcker, 1981). Reliability was assessed using composite reliability (CR) and Cronbach's alpha (α), with thresholds of 0.70 or higher indicating adequate reliability (Nunnally, 1978).

As shown in Table 3, all constructs met the recommended criteria. The AVE values ranged from 0.600 to 0.759, all exceeding the 0.50 threshold. Composite reliability coefficients ranged from 0.759 to 0.945, and Cronbach's alpha values ranged from 0.759 to 0.938, all exceeding the 0.70 threshold. These results demonstrate satisfactory convergent validity and internal consistency for all constructs.

**Table 3:** Results of the Fornell-Larcher criteria.

Variable names	Attitude	Control	Norm
Attitude	0.780		
Control	0.802	0.871	
Norm	0.669	0.769	0.775

#### 4.2.2 Discriminant validity

Discriminant validity was assessed using the Fornell-Larcker criterion and the Heterotrait-Monotrait ratio (HTMT). According to the Fornell-Larcker criterion, the square root of the AVE for each construct should exceed its highest correlation with any other construct (Fornell and Larcker, 1981). As shown in Table 3, the diagonal values (square roots of AVE) are indeed larger than the corresponding off-diagonal correlations, confirming satisfactory discriminant validity.

We further assessed discriminant validity using the HTMT ratio, with values below 0.85 indicating adequate discriminant validity (Henseler et al., 2015). All HTMT values were below the conservative threshold of 0.85, providing additional evidence of discriminant validity.

#### 4.2.3 Common method variance

We employed two approaches to assess the potential threat of common method variance (CMV). First, Harman's single-factor test was conducted; the unrotated factor solution revealed that the first factor accounted for 31.2% of the variance, well below the 50% threshold, suggesting that CMV is not a pervasive concern (Podsakoff et al., 2003).

**Figure 2:**
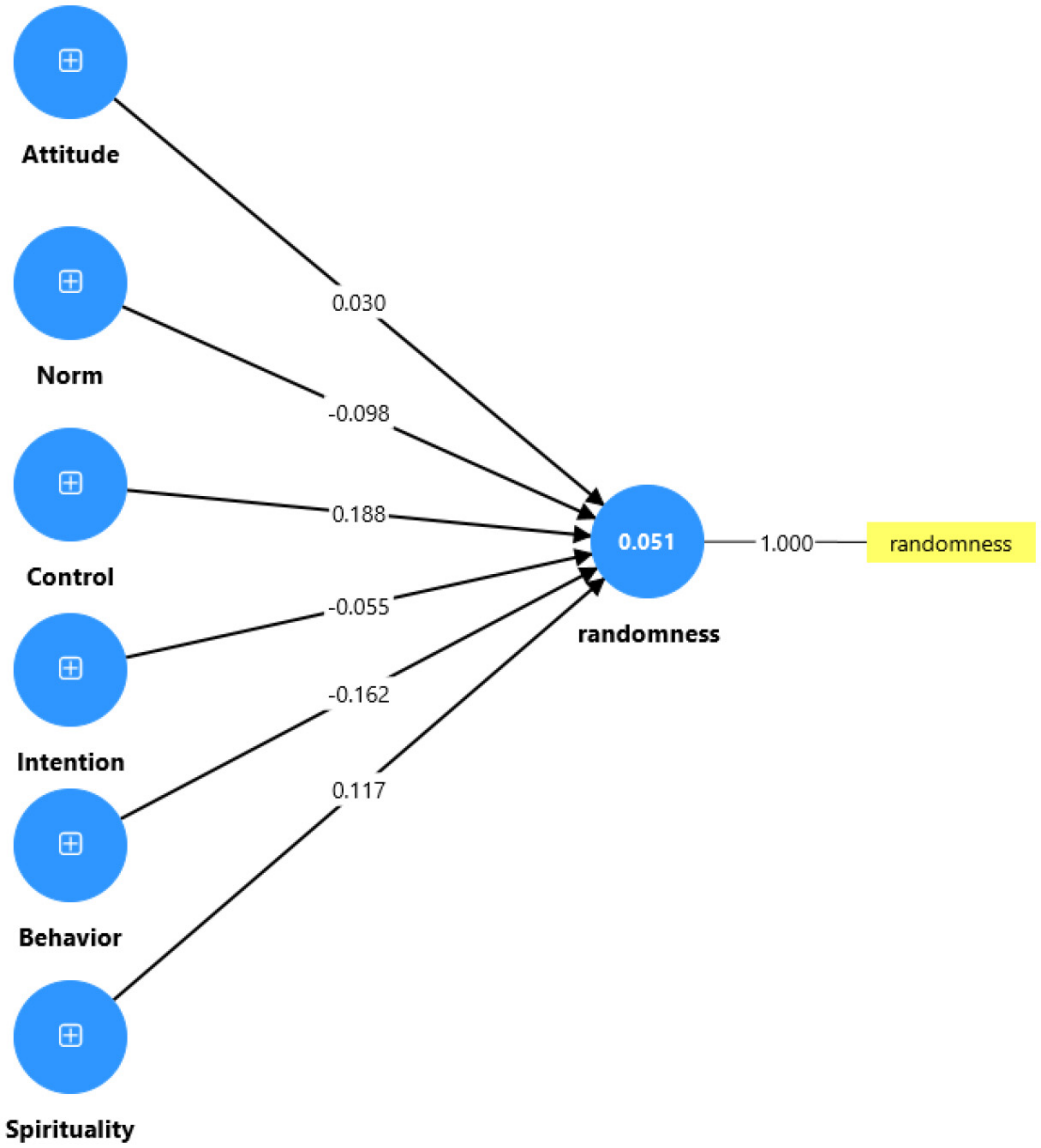
Assessing the CMV of the model.

Second, we employed the marker variable approach by including a random variable as a dependent variable in the model, treating the other constructs as independent variables (Kock, 2015). As illustrated in Figure 2, the analysis yielded path coefficients from all six substantive constructs to the marker variable. All Variance Inflation Factors (VIFs) were relatively low, ranging from 1.081 to 2.008, well below the recommended threshold of 3.3 (Hair et al., 2017). These results indicate minimal association between the substantive constructs and the marker variable, further confirming that CMV does not pose a significant threat to the validity of our findings.

### 4.3 Structural model assessment

After confirming the measurement model's adequacy, we proceeded to evaluate the structural model. This assessment included examination of path coefficients, significance levels, effect sizes (f^2^), coefficients of determination (*R*^2^), and model fit indices.

#### 4.3.1 Model fit indices

Prior to hypothesis testing, we assessed overall model fit using several indices. The normed Chi-square statistic (χ^2^/*df* ) was 1.385, well below the threshold of 3. The Standardized Root Mean Square Residual (SRMR) was 0.029, below the recommended cutoff of 0.08 (Hu and Bentler, 1999). The Root Mean Square Error of Approximation (RMSEA) was 0.042, below the 0.08 threshold. Additionally, the Comparative Fit Index (CFI) was 0.992, the Normed Fit Index (NFI) was 0.973, and the Goodness of Fit Index (GFI) was 0.968, all exceeding the recommended threshold of 0.90. These indices collectively indicate excellent model fit.

#### 4.3.2 Hypothesis testing

We tested the seven research hypotheses using PLS-SEM with bootstrapping (5,000 resamples, one-tailed). Table 4 presents a summary of the path coefficients, standard errors, *t*-values, *p*-values, and confidence intervals for all hypothesized relationships.

**Table 4:** Summary of the results of hypotheses.

Hypothesis	Path	Indicators	Result
H1	Attitude, norms, control → PET	χ^2^/*df* = 1.385, SRMR = 0.029, CFI = 0.992	Supported
H2	PET → entrepreneurial intention	**+**, **β** **=** 0.687 (*p* < 0.001), between 95% CI [0.607, 0.767]	Supported
H3	Entrepreneurial intention → preparatory behavior	**+**, **β** **=** 0.628 (*p* < 0.001), between 95% CI [0.540, 0.716]	Supported
H4	Spirituality → entrepreneurial intention	**+**, **β** **=** 0.106 (*p* = 0.024), between 95% CI [0.014, 0.198].	Supported
H5	Spirituality → preparatory behavior	**+**, **β** **=** 0.148 (*p* < 0.001), between 95% CI [0.066, 0.230]	Supported
H6	Spirituality × PET → entrepreneurial intention	**+**, β = 0.100, *p* = 0.001, between 95% CI [0.041, 0.159]	Supported
H7	Spirituality × intention → preparatory behavior	**+**, β = 0.149, *p* < 0.001, between 95% CI [0.086, 0.212]	Supported

**Figure 3:**
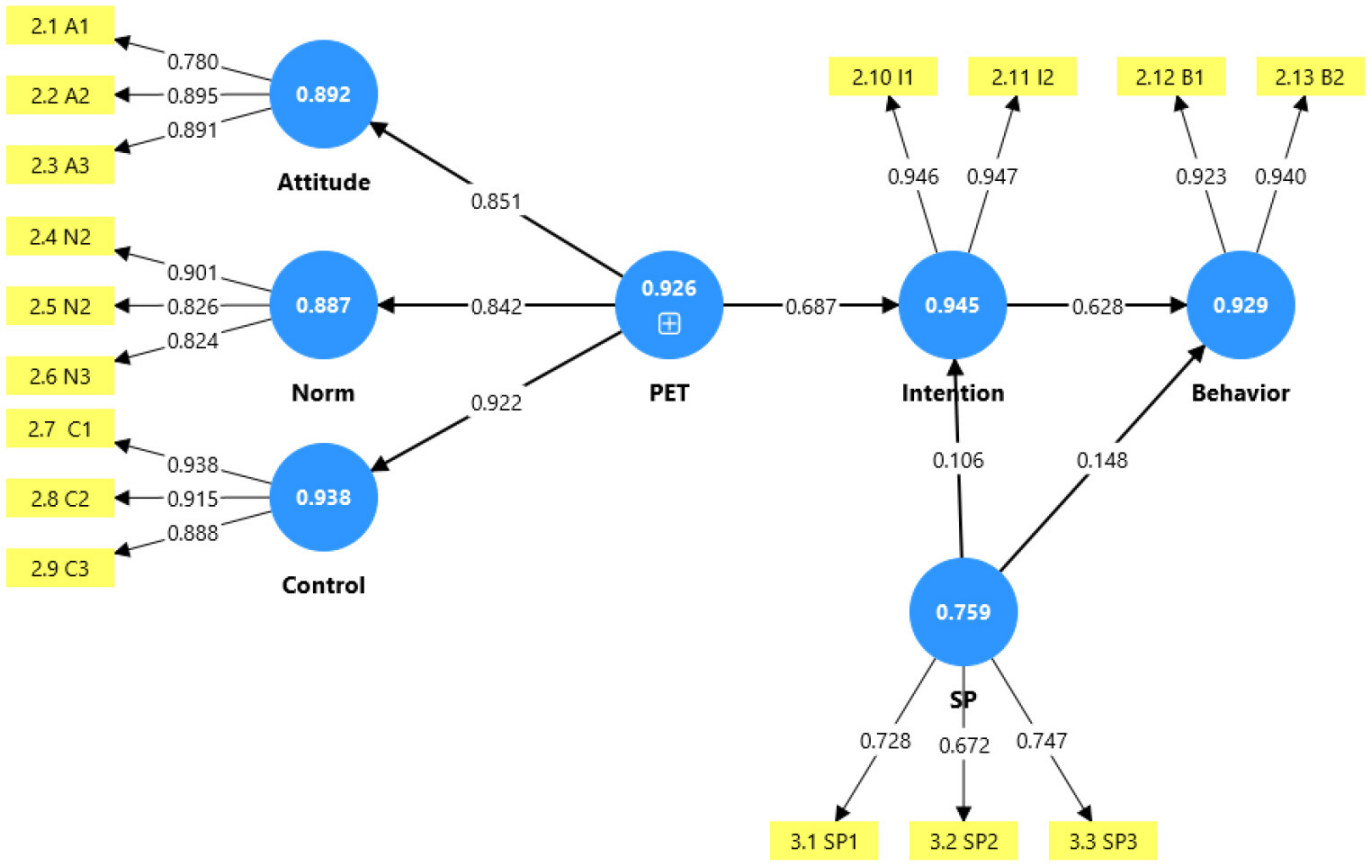
Results of path coefficients and reliability coefficients (in circles).

**H1: Formation of Planned Entrepreneurial Traits (PET)**. H1 proposed that attitude toward entrepreneurship, subjective norms, and perceived behavioral control are three dimensions forming the higher-order construct of PET. The confirmatory factor analysis results, reported in Section 4.2, demonstrated that all three dimensions loaded significantly onto the second-order construct, with factor loadings ranging from 0.842 to 0.922, as shown in Figure 3. The model fit indices for the second-order measurement model were excellent (χ^2^/*df* = 1.385, SRMR = 0.029, CFI = 0.992), providing strong support for H1. This finding also establishes the foundation for testing the predictive validity of PET in subsequent hypotheses.

**H2: PET** **→** **Entrepreneurial Intention**. H2 predicted a positive relationship between PET and entrepreneurial intention. As shown in Table 4 and Figure 3, the path coefficient was 0.687 (*p* < 0.001), with a 95% confidence interval [0.607, 0.767]. The effect size (*f*^2^ = 0.472) exceeds the threshold for a large effect (Cohen, 1988). PET explained 52.7% of the variance in entrepreneurial intention (*R*^2^ = 0.527), indicating substantial explanatory power. These results provide strong support for H2 and further confirm the predictive validity of the PET construct established in H1.

**H3: Entrepreneurial Intention** **→** **Preparatory Behavior**. H3 predicted a positive relationship between entrepreneurial intention and preparatory entrepreneurial behavior. As shown in Figure 3, the path coefficient was 0.628 (*p* < 0.001), with a 95% confidence interval [0.540, 0.716]. The effect size (*f*^2^ = 0.394) is large, and intention explained 48.3% of the variance in preparatory behavior (*R*^2^ = 0.483). Thus, H3 is supported.

**H4: Spirituality** **→** **Entrepreneurial Intention**. H4 predicted a positive direct effect of spirituality on entrepreneurial intention. As shown in Figure 3, the path coefficient was 0.106 (*p* = 0.024), with a 95% confidence interval [0.014, 0.198]. Although the effect size is small (*f*^2^ = 0.042), the relationship is statistically significant, supporting H4.

**H5: Spirituality** **→** **Preparatory Behavior**. H5 predicted a positive direct effect of spirituality on preparatory entrepreneurial behavior. As shown in Figure 3, the path coefficient was 0.148 (*p* < 0.001), with a 95% confidence interval [0.066, 0.230]. The effect size is small (*f*^2^ = 0.058), but the relationship is statistically significant, supporting H5.

#### 4.3.3 Moderation effects

**Figure 4:**
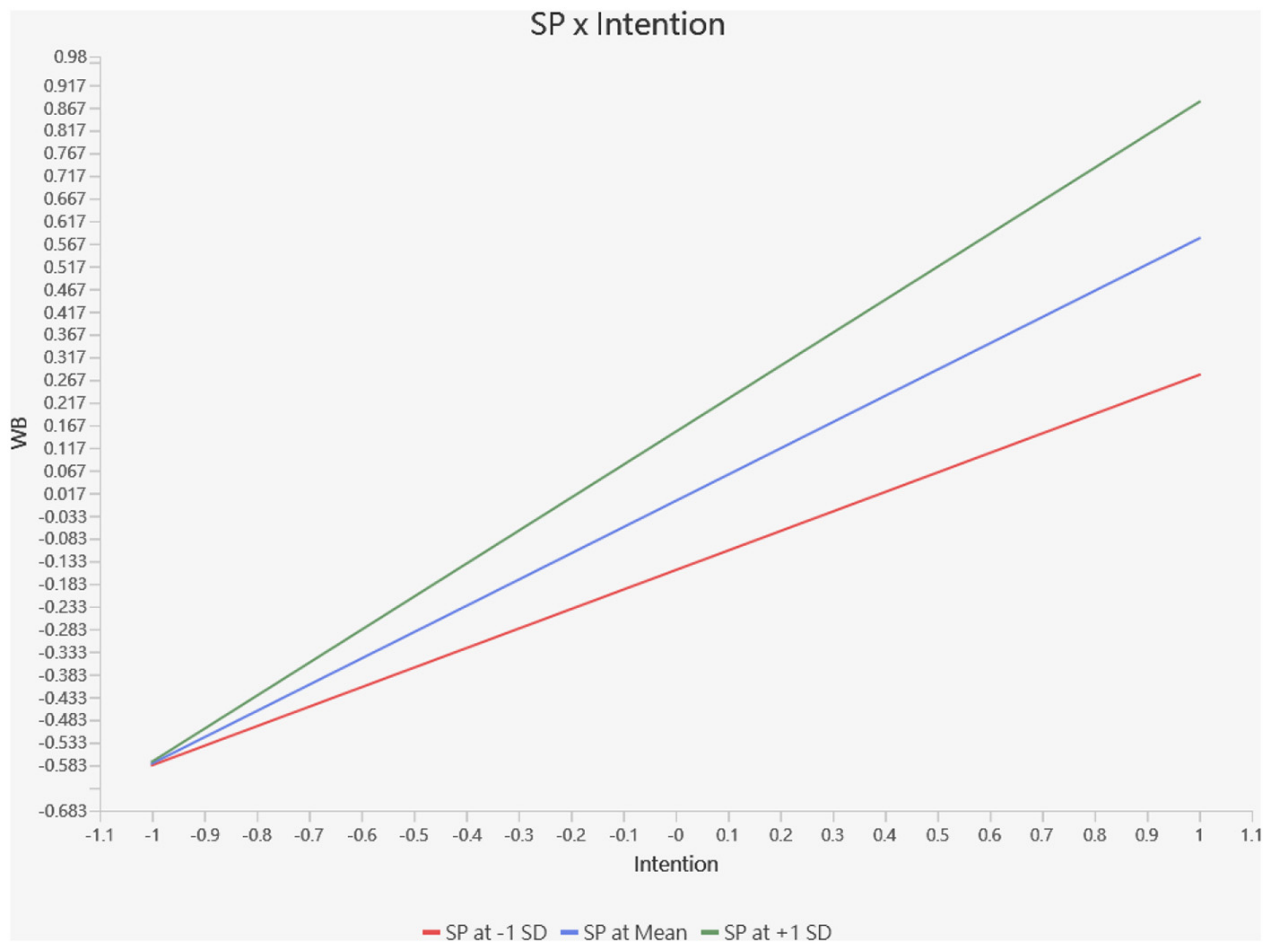
Chart 1: The simple slope analysis of spirituality for entrepreneurship on intention.

**H6: Spirituality Moderates PET** **→** **Intention**. H6 predicted that spirituality positively moderates the relationship between PET and entrepreneurial intention. The interaction term (Spirituality × PET) was significant (β = 0.100, *p* = 0.001, 95% CI [0.041, 0.159]), with a small effect size (*f*^2^ = 0.051). To interpret the nature of this moderation, we conducted a simple slope analysis, as shown in Figure 4.

As illustrated in Figure 3, the relationship between PET and entrepreneurial intention is stronger at higher levels of spirituality (+1 SD: β = 0.787, *p* < 0.001) compared to lower levels of spirituality (−1 SD: β = 0.587, *p* < 0.001). The positive slope associated with higher spirituality indicates that spiritual individuals translate their entrepreneurial traits into intention more effectively than their less spiritual counterparts. The divergence of the lines at −1 standard deviation is particularly noteworthy, suggesting that the moderating effect becomes more pronounced as PET increases. These results support H6.

**H7: Spirituality Moderates Intention** **→** **Behavior**. H7 predicted that spirituality positively moderates the relationship between entrepreneurial intention and preparatory behavior. The interaction term (Spirituality × Intention) was significant (β = 0.149, *p* < 0.001, 95% CI [0.086, 0.212]), with a small effect size (*f*^2^ = 0.062).

**Figure 5:**
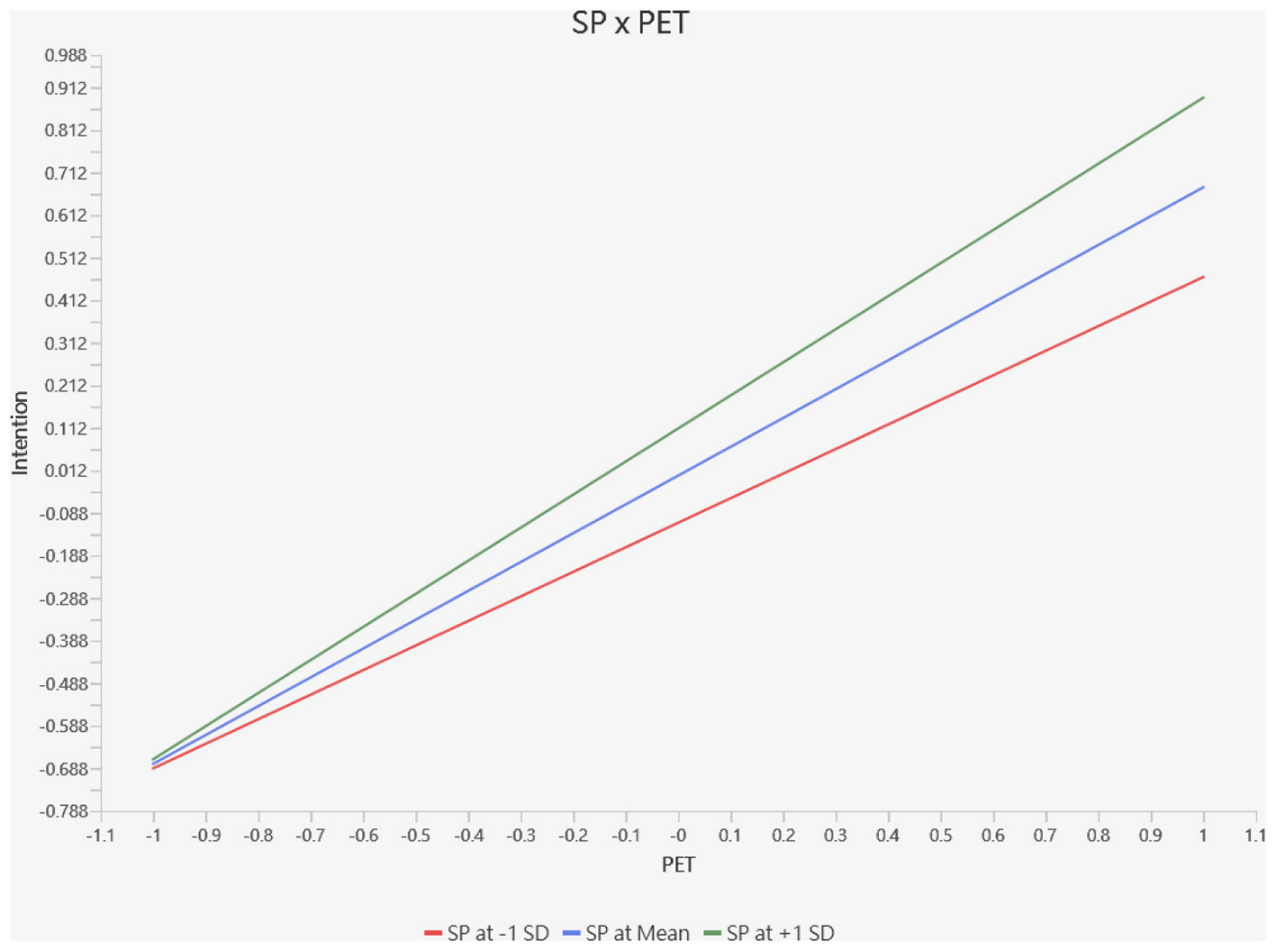
Chart 2: The simple slope analysis of spirituality for intention on behavior.

As shown in Figure 5, the positive relationship between entrepreneurial intention and preparatory behavior is stronger at higher levels of spirituality (+1 SD: β = 0.777, *p* < 0.001) compared to lower levels of spirituality (−1 SD: β = 0.479, *p* < 0.001). All three lines exhibit positive slopes, indicating that intention consistently influences behavior across varying levels of spirituality. However, as spirituality increases, the positive relationship between intention and behavior strengthens, suggesting that individuals with higher spirituality are more likely to translate their intentions into actual preparatory actions. Conversely, at lower levels of spirituality, the connection between intention and behavior weakens. These patterns underscore the importance of spirituality as a moderating factor that enhances the intention–behavior link, supporting H7.

#### 4.3.4 Endogeneity assessment

**Figure 6:**
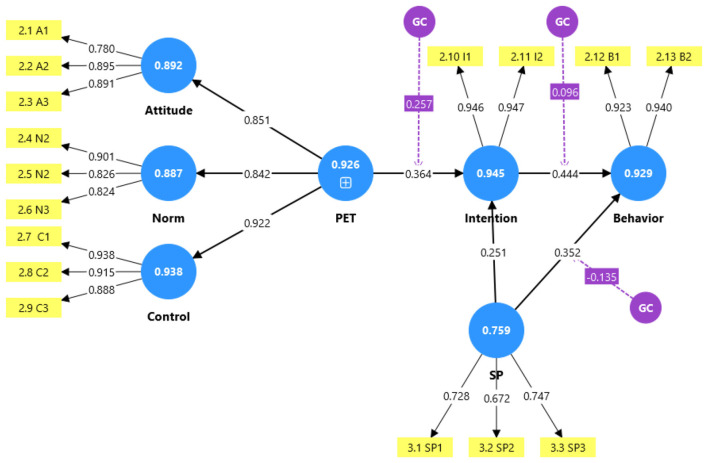
Results of the GC effects.

To strengthen causal inference for the core structural relationships (H2, H3, and H5), we employed the Gaussian Copula approach following (Park and Gupta 2012). The Gaussian Copula terms for PET (predicting intention), intention (predicting behavior), and spirituality (predicting behavior) were all non-significant (*p* > 0.10), indicating that endogeneity is not a serious concern in the estimated model. These results provide additional confidence that the identified path coefficients represent causal effects rather than spurious correlations. As shown in Figure 6, Gaussian Copula results are shown, i.e. PET-> Intention is significant as GC > 0.1 and SP -> Behavior also is significant as GC > 0.1.

### 4.4 Summary of results

All seven hypotheses received empirical support. The structural model explained substantial variance in both entrepreneurial intention (*R*^2^ = 0.527) and preparatory entrepreneurial behavior (*R*^2^ = 0.483). Effect sizes for the core TPB relationships (H2 and H3) were large, while those involving spirituality (H4–H7) were small but statistically significant. Table 4 provides a summary of the hypothesis testing results.

## 5 Discussion

### 5.1 Summary of findings

This study investigated the role of spirituality within an extended Theory of Planned Behavior (TPB) framework in the South Korean entrepreneurial context. The empirical analysis yielded support for all seven hypothesized relationships, providing a comprehensive understanding of how spirituality interacts with entrepreneurial cognition and action.

First, the results confirmed that Planned Entrepreneurial Traits (PET)—conceptualized as a higher-order construct comprising attitude toward entrepreneurship, subjective norms, and perceived behavioral control—significantly predict entrepreneurial intention (H2: β = 0.687, *p* < 0.001). This finding aligns with meta-analytic evidence on the TPB's predictive validity in entrepreneurship (Schlaegel and Koenig, 2014) while extending it by demonstrating that the three antecedents can be meaningfully modeled as a reflective second-order construct. Entrepreneurial intention, in turn, strongly predicted preparatory entrepreneurial behavior (H3: β = 0.628, *p* < 0.001), consistent with prior research on the intention-behavior link (Kautonen et al., 2015; Sheeran, 2002).

Second, spirituality demonstrated significant direct effects on both entrepreneurial intention (H4: β = 0.106, *p* = 0.024) and preparatory behavior (H5: β = 0.148, *p* < 0.001). These findings confirm that spirituality functions as a distal antecedent within the extended TPB framework, influencing both the formation of intentions and the execution of entrepreneurial actions. The relatively small effect sizes (*f*^2^ = 0.042 and 0.058, respectively) suggest that spirituality operates as a supplementary rather than primary driver, complementing the core TPB mechanisms.

Third, and most notably, spirituality emerged as a significant moderator of two critical pathways. Drawing on Cognitive-relational Theory (Lazarus and Folkman, 1984), the relationship between PET and entrepreneurial intention was strengthened at higher levels of spirituality (H6: β = 0.100, *p* = 0.001), indicating that spiritual individuals translate their entrepreneurial traits into intentions more effectively because spiritual beliefs provide cognitive resources for reframing challenges as meaningful opportunities. More importantly, spirituality moderated the intention-behavior relationship (H7: β = 0.149, *p* < 0.001), with simple slope analysis revealing that the positive effect of intention on behavior is substantially stronger for individuals with high spirituality (+1 SD: β = 0.777) compared to those with low spirituality (−1 SD: β = 0.479). From a Social Identity Theory perspective (Tajfel and Turner, 2001), spiritual communities provide normative support and accountability that help bridge the gap between intention and action. This finding directly addresses the well-documented intention-behavior gap in entrepreneurship research.

The structural model explained substantial variance in both entrepreneurial intention (*R*^2^ = 0.527) and preparatory behavior (*R*^2^ = 0.483), indicating strong explanatory power. Table 4 provides a consolidated summary of the hypothesis testing results.

### 5.2 Theoretical implications

This study makes several contributions to entrepreneurship theory and the extended TPB literature. First, our findings extend the TPB by integrating spirituality as both an antecedent and a moderator within a unified framework. While prior extensions of the TPB have incorporated variables such as personality traits (Sonkar et al., 2026), emotions, and experience, the role of deeply held personal belief systems has remained underexplored. The present study demonstrates that spirituality significantly enhances the explanatory power of the TPB, particularly in understanding how intentions translate into action. By drawing on Self-Determination Theory, Cognitive-relational Theory, and Social Identity Theory, we provide a multi-theoretical account of how spirituality operates—fulfilling intrinsic needs for meaning, reframing challenges as growth opportunities, and leveraging community support to sustain action. This contributes to a more holistic understanding of entrepreneurial motivation that moves beyond purely rational-cognitive models to encompass existential and meaning-based dimensions of human behavior (Smith et al., 2023; Rashid and Ratten, 2022).

Second, the findings contribute to the literature on the intention-behavior gap in entrepreneurship. Meta-analytic evidence indicates that intention explains approximately 28% of the variance in behavior, leaving substantial room for moderating influences (Sheeran, 2002). The present study identifies spirituality as a significant bridging mechanism that strengthens the intention-behavior relationship. The observed moderating effect (β = 0.149) indicates that spiritual individuals are more likely to act on their entrepreneurial intentions, reducing the gap between planning and action. This finding advances theoretical understanding of why some individuals successfully initiate venture creation while others, despite strong intentions, fail to take preparatory steps. The mechanisms underlying this effect—enhanced commitment through self-concordant goal pursuit (Ryan and Deci, 2000), emotional regulation and resilience through cognitive reappraisal (Lazarus and Folkman, 1984), and social accountability from spiritual communities (Balog et al., 2014)—provide a theoretical foundation for future research on how personal belief systems facilitate goal attainment.

Third, this study contributes to the cross-cultural entrepreneurship literature by empirically validating the extended TPB model in a non-Western, collectivist context. While prior research has often treated culture as a monolithic variable, we demonstrate that spirituality operates through both universal psychological mechanisms (intrinsic motivation, cognitive reappraisal, social identification) and culturally specific expressions. The results demonstrate that spirituality operates through culturally specific mechanisms in South Korea. The influence of subjective norms, for instance, is likely amplified by the Korean concept of *jeong* (deep relational attachment), while the effect of perceived behavioral control may be enhanced by *han* (collective resilience in overcoming adversity) (Cho, 2009). By situating the investigation in South Korea, this study responds to calls for greater attention to context-specific psychological mechanisms in entrepreneurial behavior research (Welter, 2011) and provides a foundation for cross-cultural comparisons.

Fourth, the conceptualization and validation of Planned Entrepreneurial Traits (PET) as a second-order construct represents a methodological contribution. By modeling the three TPB antecedents as indicators of a higher-order construct, this study achieves theoretical parsimony while preserving the integrity of the underlying dimensions (Becker et al., 2012). The strong predictive validity of PET (β = 0.687 toward intention, compared to the meta-analytic average of β = 0.45 for the three separate antecedents reported by Schlaegel and Koenig, 2014) suggests that the second-order approach captures synergistic effects that may be obscured when examining the dimensions individually. This finding has implications for future TPB research, particularly in contexts where the three antecedents are expected to operate in concert.

Finally, this study contributes to the emerging literature on spirituality and entrepreneurship by providing quantitative evidence from a large-sample study. While prior research has largely been qualitative or conceptual (Dodd and Seaman, 1998; Shepherd et al., 2024), the present study offers empirical validation of spirituality's role using rigorous psychometric methods. The finding that spirituality enhances both the formation and execution of entrepreneurial intentions, and that these effects are robust even among non-religious respondents, provides a foundation for integrating spirituality into mainstream entrepreneurship theory.

### 5.3 Practical implications

The findings of this study have several practical implications for entrepreneurship education, training programs, and policy in South Korea.

The results suggest that entrepreneurship curricula should be expanded beyond traditional business and technical skills to incorporate elements that help students explore their personal values, sense of purpose, and spiritual beliefs. Given the cultural resonance of concepts such as *jeong* and *han* in Korea, these programs should be culturally adapted to resonate with students' lived experiences. For example, incorporating discussions of how entrepreneurial challenges can be reframed as opportunities for collective perseverance (*han*) or how business relationships can embody deep relational commitment (*jeong*) may enhance the relevance and effectiveness of such interventions.

Beyond formal education, entrepreneurial support organizations and mentoring programs should consider incorporating components that foster resilience, wellbeing, and value clarification. Training modules that help aspiring entrepreneurs develop emotional regulation skills—through meditation, reflective practices, or peer support groups—could strengthen their ability to persist through the inevitable setbacks of venture creation by leveraging the cognitive reappraisal mechanisms described in Cognitive-relational Theory (Lazarus and Folkman, 1984). Mentors should be trained to recognize and discuss the role of personal values and spiritual beliefs in sustaining entrepreneurial motivation, creating a safe space for mentees to explore these dimensions of their entrepreneurial journey.

The Korean government's K-Startup initiatives and other entrepreneurship support programs could benefit from a more holistic approach to fostering entrepreneurial activity. While current policies focus primarily on financial support, incubation infrastructure, and technical training, the findings of this study suggest that attending to the personal belief systems of young entrepreneurs (target age 18–25 years, consistent with our sample) could enhance the effectiveness of these investments. Policies that support the integration of mindfulness and wellbeing components into entrepreneurship programs, or that fund research on value-driven entrepreneurship, could yield significant returns by helping more aspiring entrepreneurs translate their intentions into sustained preparatory action.

Individual entrepreneurs may draw practical insights from this research by intentionally cultivating spiritual practices or reflection on personal values as a resource for navigating the entrepreneurial journey. Recognizing that entrepreneurship is not merely an economic activity but can be experienced as a calling or a path to self-actualization may provide a deeper source of motivation that sustains effort through difficulties. Engaging with spiritual communities, whether religious congregations, meditation groups, or other communities of shared values, can provide social support, accountability, and encouragement that facilitate translating intentions into action.

### 5.4 Limitations

This study has several limitations that should be considered when interpreting the findings.

The data were collected at a single point in time, which limits the ability to make causal inferences about the relationships among variables. While the theoretical model posits directional relationships (e.g., PET → intention → behavior), the cross-sectional design cannot definitively establish temporal precedence or rule out reverse causality. Longitudinal research tracking individuals over time is needed to strengthen causal claims and to observe how the relationships among spirituality, intention, and behavior evolve through the venture creation process.

The sample consisted of 204 university students and recent graduates from a single large university in Seoul. While this population is appropriate for studying entrepreneurial intentions and preparatory behavior—as students are at a career stage where entrepreneurial pathways are actively considered—the findings may not generalize to established entrepreneurs, who face different challenges and constraints. Moreover, the sample's demographic characteristics (mean age 22.3 years, predominantly undergraduate students) limit generalizability to older entrepreneurs or those with different educational and professional backgrounds.

All constructs were measured using self-report scales, which are susceptible to social desirability bias and common method variance. Although statistical tests suggested that common method bias is not a serious concern, the possibility remains that respondents' answers were influenced by their desire to present themselves favorably, particularly regarding socially valued characteristics such as spirituality and entrepreneurial motivation.

The spirituality scale employed in this study (Lee, 2024) has been used in Korean samples but requires further validation to establish its cultural specificity relative to Western spirituality measures. The scale items assess generic dimensions of spirituality (search for meaning, sense of connection, value-driven orientation) that may not fully capture culturally specific Korean spiritual constructs such as *jeong* and *han*. Future research should develop and validate measures that more directly assess both universal and culturally embedded spiritual concepts.

Entrepreneurial behavior was operationalized as preparatory actions toward venture creation (e.g., business planning, resource seeking, networking) rather than actual business establishment or long-term venture success. While this focus is appropriate for a student sample, it limits the ability to draw conclusions about spirituality's role in venture survival, growth, and performance over time.

This study focused on the positive moderating role of spirituality, but emerging research suggests that spirituality may also have negative manifestations in entrepreneurship, including spiritual bypass (using spiritual beliefs to avoid practical challenges), excessive rigidity in adhering to spiritual principles, and burnout from overcommitment to spiritually motivated ventures (Shepherd et al., 2024). The present study did not investigate these potential negative effects, representing an important direction for future research.

### 5.5 Future research directions

Building on the findings and limitations of this study, several avenues for future research are proposed.

Future research should employ longitudinal designs tracking individuals over 12 to 24 months to examine how the relationships among spirituality, intention, and behavior unfold through the venture creation process. Such designs would enable stronger causal inferences and allow observation of how spirituality influences persistence through different stages of entrepreneurial activity. Longitudinal research could also examine whether the moderating effect of spirituality on the intention-behavior relationship strengthens or weakens over time as individuals encounter different types of challenges.

While this study was deliberately situated in South Korea to examine culturally specific mechanisms, future research should systematically investigate how the role of spirituality in entrepreneurship varies across cultural contexts. Cross-cultural replication studies in other collectivist Asian societies (e.g., China, the Philippines, Indonesia) would help distinguish universal psychological mechanisms from culture-specific manifestations. Research in individualistic Western contexts would test whether spirituality operates similarly when subjective norms are less collectivist and when spiritual expression is more privatized. Such cross-cultural comparisons would refine understanding of the boundary conditions of the extended TPB model.

Also, future research should develop and validate measures that more directly assess culturally specific spiritual constructs in the Korean context. Such measures would enable more precise tests of how indigenous spiritual concepts influence entrepreneurial processes. Additionally, multi-method research combining self-report scales with behavioral measures (e.g., observed preparatory actions, archival data on venture registration) would reduce concerns about common method bias and provide more objective indicators of entrepreneurial behavior.

While this study focused on preparatory behavior, future research should examine whether spirituality predicts longer-term entrepreneurial outcomes, including venture survival, growth, innovation, and profitability. Theoretical arguments suggest that spirituality may foster the resilience, ethical grounding, and long-term vision needed to sustain ventures through difficulties (Shepherd et al., 2024), but empirical evidence is needed to test these propositions. Longitudinal research tracking ventures from inception through early growth and, potentially, exit would provide valuable insights into spirituality's role across the entrepreneurial lifecycle.

Finally, future research should explore how the extended TPB with spirituality can be integrated with other theoretical perspectives on entrepreneurship. Connections to entrepreneurial identity theory (how spirituality shapes identity as an entrepreneur), effectuation theory (how spiritual values influence means-driven rather than goal-driven reasoning), and sustainable entrepreneurship (how spirituality fosters triple-bottom-line orientation) represent promising avenues for theoretical integration and empirical investigation.

## Data Availability

The raw data supporting the conclusions of this article will be made available upon request to the authors.

## Appendix

**Appendix A: Survey Measurement Items**

All items were measured on a 6-point Likert scale (1 = Strongly Disagree, 6 = Strongly Agree).

**A.1 Theory of Planned Behavior (TPB) Constructs**

*(Source: Adapted from Ajzen*, *1991**)*

**Attitude (3 items)**

1. I believe starting a business is a good career choice.
2. I think being an entrepreneur would give me great satisfaction.
3. The idea of creating a new business excites me.

**Subjective Norm (3 items)**

1. People important to me would approve if I started a business.
2. My family supports the idea of me becoming an entrepreneur.
3. My friends think that starting a business is a good idea.

**Perceived Behavioral Control (3 items)**

1. I am confident in my ability to start and run a business.
2. I can effectively solve problems that arise in business.
3. I have the necessary skills to start a business.

**A.2 Entrepreneurial Intention (2 items)**

*(Source: Adapted from Liñán and Chen*, *2009**)*

1. I intend to start a business in the near future.
2. I am actively making plans to start a business.

**A.3 Preparatory Entrepreneurial Behavior (2 items)**

*(Source: Adapted from Kautonen et al.*, *2015**)*

1. I am currently involved in starting a business.
2. I regularly engage in activities that are related to starting a business.

**A.4 Spirituality (6 items)**

*(Source: Adapted from Lee*, *2024**)*

1. I believe my life has meaning and purpose.
2. I maintain my inner peace whatever happens.
3. I attend sessions, workshops, etc., that are focused on spirituality or religion.
4. I accept that I am not in full control of the course of my life.
5. I feel strongly connected when I am in nature.
6. I have had experiences in which all things seemed part of a greater whole.
